# Response to the Netflix Docuseries “Big Vape: The Rise and Fall of JUUL”: Mixed Methods Analysis of YouTube Comments Using Qualitative Coding and Topic Modeling

**DOI:** 10.2196/76737

**Published:** 2025-09-19

**Authors:** Beth Hoffman, Arpita Tripathi, Ariel Shensa, Julia (Pengyue) Dou, Piper Narendorf, Nishi Hundi, Jaime Sidani

**Affiliations:** 1 Department of Behavioral and Community Health Sciences, School of Public Health, University of Pittsburgh, 130 De Soto Street, Pittsburgh, PA, 15201, United States, 1 4126245859; 2 Department of Health Administration and Public Health, John G Rangos Sr. School of Health Sciences, Duquesne University, Pittsburgh, PA, United States

**Keywords:** nicotine and tobacco products, social media data, e-cigarettes, JUUL, misinformation

## Abstract

**Background:**

On October 11, 2023, Netflix released the docuseries “Big Vape: The Rise and Fall of JUUL,” which chronicled the founding of JUUL, its rise in popularity among youth, and the subsequent public backlash. The official Netflix YouTube channel posted a trailer promoting the docuseries and an official clip from the docuseries. Recent studies have demonstrated the utility of using comments posted under YouTube videos to analyze reactions to the content and discourse around the health topics explored in the video.

**Objective:**

This study aimed to (1) systematically characterize nicotine and tobacco product (NTP)–related comments and replies posted in response to the docuseries trailer and video clip and (2) explore integration of automated topic modeling techniques with traditional human-generated qualitative coding.

**Methods:**

We extracted all comments and replies on the aforementioned YouTube clips 1 month after the docuseries’ release (N=532). Research assistants manually double-coded the comments using a systematically developed codebook that assessed for NTP sentiment (pro-NTP, anti-NTP, complex sentiment, or no sentiment) and the presence or absence of specific electronic cigarette (e-cigarette)–related content. Given the substantial amount of comments coded as potential misinformation during the coding process, we conducted an in-depth qualitative content analysis of all comments coded as potential misinformation. Simultaneously, we used word clustering techniques including structural topic modeling to identify the overarching topics.

**Results:**

Of the 73.8% ( 393/532) relevant comments, 63.6% (250/393) expressed NTP sentiment with 42.8% of these (107/250) expressing pro-NTP sentiment and 18.4% (46/250) expressing complex sentiment. The most frequent content category was potential misinformation (27.5%, 108/393). These 108 comments contained 152 individual pieces of misinformation that were broadly grouped within 6 themes with various numbers of subthemes; the most frequent misinformation theme was that e-cigarette use is completely safe or much safer than smoking (n=80). Other frequently occurring content categories included e-cigarette use is safer than smoking (17.6%, 69/393), and personal experience using e-cigarettes or JUUL (15.5%, 61/393). For topic modeling, we identified 9 topics that we qualitatively assigned into 4 thematic categories: comparisons with other drugs, mentions of government and pharma companies, role of media and parents, and harms associated with nicotine and tobacco products.

**Conclusions:**

To the best of our knowledge, this is the first study to examine viewer reactions to the docuseries about JUUL. Our analysis of YouTube comments offers insight into current sentiment and misinformation regarding NTPs and highlights the potential utility of using mixed methods to analyze NTP-related social media data, and the benefits of integrating computational and human qualitative research to analyze social media perceptions of e-cigarettes. Public health professionals can use our findings to help develop tailored health communication messages to address common sentiment and misconceptions related to JUUL, other e-cigarette products, and new NTP products.

## Introduction

In 2018, the U.S. Surgeon General declared an epidemic of youth electronic cigarette (e-cigarette) use, citing the 78% increase in current e-cigarette use among high school students and 48% increase among middle school students from 2017 [1]. The Surgeon General’s advisory noted that this increase was largely driven by use of the brand JUUL, which by the end of 2017 held the greatest market share in the United States of all e-cigarette products [1].

JUUL, an electronic nicotine delivery system (ENDS), delivers a substantial amount of nicotine, with each “vape pod” containing an amount equivalent to a pack of cigarettes [2]. It quickly became synonymous with e-cigarette use due to its sleek design, high nicotine content, and appealing flavors [3], and studies highlighted public health issues such as nicotine addiction and respiratory problems associated with JUUL use [3,4]. In response to the condemnation from regulators, parents, and health advocates, JUUL voluntarily withdrew its flavored products from the market in 2019 [5]. This move aimed to address concerns about the appeal of flavored e-cigarettes to youth and to further reduce their access to these products.

In July 2019, a widespread outbreak of e-cigarette or vaping product use–associated lung injury (ie, EVALI) garnered significant attention among medical professionals, the news media, and the public. The US Centers for Disease Control and Prevention (CDC) coordinated a multistate outbreak investigation, with over 2800 cases requiring hospitalization reported to the organization by February 18, 2020 [6]. Ultimately, most cases were linked to informally sourced (ie, “black market”) products that contained tetrahydrocannabinol (THC), although about 20% (227/956) of affected individuals who reported using any nicotine-containing product reported using nicotine-containing e-cigarettes only [6].

In December 2019, Congress passed the Tobacco 21 (T-21) law, which aimed to restrict the sale of tobacco and e-cigarette products, including JUUL, to individuals aged 21 and older [7]. On January 2, 2020, the Food and Drug Administration (FDA) issued a ban on the sale of flavored JUUL pods, except for tobacco and menthol flavors [8]. Further, in 2022, the FDA imposed stricter regulations by mandating JUUL to submit extensive premarket tobacco product applications (PMTAs) to remain on the market; weeks later, the FDA denied these PMTAs, citing insufficient data on health risks, which led to a temporary ban on JUUL products [9]. Most recently, on June 6, 2024, the FDA announced that it has lifted its ban as the agency reviews new court rulings and evaluates updated information submitted by the vape manufacturer [10]. Per the 2024 National Youth Tobacco Survey, JUUL is the fifth most popular e-cigarette brand used by youth, behind disposable e-cigarettes Elf Bar, Breeze, Mr. Fog, and Vuse; these have become more popular among youth due to their variety of flavors and lower cost [11].

On October 11, 2023, the streaming service Netflix released a docuseries titled “Big Vape: The Rise and Fall of JUUL.” This 4-episode docuseries examined the founding of JUUL, the product launch, aggressive marketing tactics, regulatory challenges, and the subsequent public backlash [12]. Previous research has examined viewer reactions to EVALI storylines on popular medical dramas [13,14], as well as the influence of health-focused documentaries on viewer knowledge and attitudes [15]. However, to the best of our knowledge, viewer reactions to this docuseries have yet to be examined.

Social media data may provide an innovative avenue to explore this. Prior work using the social media platform X (previously named Twitter) has demonstrated that these data provide insight on viewer responses to Netflix documentaries, sentiment around e-cigarettes, personal or proximate use of e-cigarettes, and e-cigarette–related misinformation [16-22]. However, in 2023, X ceased free access to its application programming interface (API), limiting the utility of using messages on the platform for research purposes [23].

On September 21, 2023, the official Netflix YouTube channel posted a trailer promoting the docuseries, followed by posting an official clip from the docuseries on October 11, 2023. Recent studies have demonstrated the utility of using comments posted under YouTube videos to analyze reactions to the content and discourse around the health topics explored in the video [24,25], including analyzing comments posted in response to e-cigarette modification YouTube videos [26]. Similarly, analyzing comments on vaccination videos has provided insights into public perceptions, the spread of misinformation, and the effectiveness of health communication strategies [27]. These studies demonstrate that YouTube comments can directly reveal nuanced viewer reactions, engagement levels, and the spread of information or misinformation.

Automated techniques are becoming increasingly popular for analyzing social media data due to their ability to efficiently process large volumes of unstructured text and uncover underlying patterns and themes [28]. Topic modeling is one such automated method that uses machine learning to identify latent themes within textual data by analyzing word frequency and co-occurrence patterns. Unlike manual thematic coding, which requires predefined categories and can be time-consuming, topic modeling offers an inductive approach that uncovers topics directly from the data without prior assumptions [29]. This makes it especially useful for exploratory analysis, where the goal is to discover the underlying structure of conversations. When combined with human coding, topic modeling can guide researchers to areas of interest for further qualitative investigation, ensuring a comprehensive and nuanced understanding of social media discourse [30].

Therefore, we aimed to systematically characterize nicotine and tobacco product (NTP)–related comments and replies posted in response to the docuseries trailer and video clip on the Netflix YouTube channel. A secondary aim was to explore the integration of automated topic modeling techniques in comparison to traditional human-generated qualitative coding to determine whether computational methods can complement the process of human coding.

## Methods

### Data Collection

We extracted all comments and replies on the docuseries trailer and docuseries clip on YouTube 1 month after the docuseries release (November 11, 2023). This time frame was chosen given our focus on initial reactions to the docuseries, as well as a previous study that used a 1-month time frame for assessing viewer reactions to the medical drama Code Black and data suggesting that most people watch a new Netflix show soon after it becomes available [31,32]. We used a Python (Guido van Rossum) script with an API key to automate the downloading of all corresponding comments for the 2 videos. This approach enabled us to efficiently retrieve and archive the entire content and discussion data for further analysis. Comments and replies were organized into 2 spreadsheets: one for comments and replies from the trailer (n=437) and one for comments and replies from the clip (n=95). This resulted in 239 original comments with 293 associated replies, for 532 total comments and replies.

### Human Qualitative Coding

For human qualitative coding, we adapted methods previously used for codebook development and analysis of JUUL and e-cigarette–related Twitter data [21,22].

#### Codebook Development and Coding Procedures

The lead researcher for this study, first author BLH, oversaw codebook development and coding procedures. The first author and 2 research assistants (RAs) each drafted an initial list of potential codes using a hybrid process consisting of codes from prior qualitative analyses of e-cigarette–related social media data [19,21,22] and an initial analysis of 50 randomly selected comments or replies from the dataset. The first author and the 2 RAs then met to review their lists of potential codes and used these to draft an initial codebook with codes, subcodes, definitions, and examples (Multimedia Appendix 1).

Each comment or reply was initially coded for relevance to NTPs, which was defined as “A comment that mentions JUUL or NTPs, including cigarettes. It does not need to be explicit.” Comments or replies that were coded as not relevant and excluded from further analysis included ones that mentioned the docuseries but not NTPs (eg, “It’s rare to see such a well-structured criticism of Australia’s public policy. Kudos to Netflix.”) and comments or replies that mentioned substances other than NTPs (eg, “When are we going to get a documentary that shows why people should not use marijuana or drink alcohol?”). For the replies, RAs were instructed to consider the relevance of the parent comment when determining relevance. For example, the reply “saved countless lives” was coded as relevant because it was in direct response to the relevant parent comment of “Introducing vapes was a really bad idea. Now all kids in school have it.*”*

All relevant comments or replies (henceforth simply referred to as “comments”) were then coded for NTP sentiment. Comments could be coded as pro-NTP, anti-NTP, complex sentiment, or no sentiment. Comments were coded as complex sentiment if they expressed pro-e-cigarette sentiment but anti-JUUL sentiment or vice versa (eg, “I’ve never used and never trust Juul or any pre-filled vape pens. I am still good with my mod+ tank setup from the 2010s and get my PG/VG in bulk online. Honestly, I don’t even like pod systems.”) or if they expressed the complexity of evidence around the safety or harms of e-cigarette use (eg, “Vaping has been (and still is) an effective way to quit smoking, but some big companies saw the money potentials and exploited it by targeting kids with pre-filled vape pens.”). Comments were coded as no sentiment if they contained a statement with no clear sentiment or if the RAs could not determine what type of sentiment was expressed (eg, “have you seen a Juul? They look almost exactly like USB drives.”).

Next, comments were coded for the presence or absence of specific e-cigarette–related content. Content codes that emerged from direct analysis of the data included distrust of medicine, research, or government; comparison to opioids or alcohol; conspiracy; Big Tobacco; blaming parents; and more research is needed. All other content codes were adapted from prior research examining e-cigarette–related social media data [19,21,22,33]. For a complete list of codes, definitions, and examples, please see Multimedia Appendix 1.

Comments that were not written in English were entered into Google Translate for translation and coding in English. Content codes were not mutually exclusive. Following codebook development, 4 RAs were paired up (2 teams of 2) for coding. The first author oversaw all coding assignments and procedures. All comments or replies were double-coded. After coding 100 comments or replies, each team met with the first author to adjudicate differences and refine the codebook as needed. Prior to adjudication meetings, percent agreement ranged from 71%to 100%and Cohen kappa from 0.09 to 1.00; all differences were resolved during adjudication (ie, 100% agreement was reached for all categories). This was repeated until all coding was complete.

#### Analyses

We calculated descriptive statistics for each sentiment and content code and examined the distribution of content code frequency by sentiment. Given the substantial amount of comments coded as potential misinformation during the coding process, following the completion of the initial human qualitative coding, we decided to conduct an in-depth qualitative content analysis of these comments [34]. To conduct this, the first and last authors independently analyzed all comments coded as potential misinformation. First, they identified all specific comments that made statements about ENDS, JUUL, health claims, and claims related to policy. All statements were compared with the peer-reviewed literature (eg, statements about harms and benefits) or policy documents and news sources (eg, statements about policy) as of March 2024. It is important to note that, in many cases, there has not yet been a definitive consensus regarding health claims in the peer-reviewed literature, meaning that articles exist that both “prove” and “disprove” a particular statement. Because a scientific consensus has not yet been reached, these claims were categorized as misinformation. In some cases, this includes exaggeration of research findings. Then, the first and last authors tabulated the total number of phrases identified as misinformation, organized these phrases into overarching misinformation categories, and identified common words within each category.

### Topic Modeling

Simultaneously, we used R (R Core Team) to perform topic modeling with all comments and replies. Topic modeling is a text-analysis method that identifies groups of co-occurring terms or latent topics in large bodies of text, often referred to as “corpus.” In this study, the text corpus was the set of comments and replies described earlier. Topic modeling in this study was done in 2 main steps: we first used R packages, including tidytext, dplyr, stringr, and stopwords for data cleaning and text preprocessing. This involved converting the text to lowercase, removing punctuation, and filtering out stop-words. Posttext cleaning, we generated a frequency analysis of the most frequently used terms and created a Document-Term Matrix using the tm package. Later, topic modeling was performed using latent Dirichlet allocation (LDA), which is the most commonly used algorithm for topic modeling [35], and has been used in previous studies using social media data for health-based topics [36-38]. We explored the LDA-based model with various topic numbers, including k=10, 9, 8, and 5, based on recommendations by previous studies [35]. The final analysis focused on models with 10 topics. After reviewing the content of the k=5, 8, 9, and 10 solutions, the first, second, and last authors (BLH, AT, and JES) determined that the 10-topic model provided the most coherent and qualitatively meaningful representation of the data. To assess robustness, we repeated models with multiple random seeds and bootstrap resamples, confirming stable topic assignments. For benchmarking, we additionally implemented non-negative matrix factorization (NMF) and BERTopic (embedding-based clustering) and compared results on coherence, exclusivity, and stability. Topics were pruned if they were predominantly non-English or qualitatively uninterpretable (eg, use of emoticons). One such topic was removed, and the remaining 9 topics were grouped inductively into 4 thematic categories for reporting.

### Ethical Considerations

This study was approved by the University of Pittsburgh Human Research Protection Office (approval number: STUDY22080079). As this study only involved the examination of publicly available social media data, informed consent and participant compensation were not applicable. Regarding privacy, although all comments included in our sample are publicly available, we recognize the sensitive nature of some comments, particularly those where a user self-disclosed using NTPs. Therefore, consistent with previous analyses of publicly available NTP content on social media [20-22], we modified all direct quotes in the tables and text and conducted reverse searches (ie, inputting the modified text into Google) to ensure that the modifications are sufficient to prevent individual users from being identified. In the absence of specific reporting guidelines related to social media research, we followed the SRQR (Standards for Reporting Qualitative Research) [39].

## Results

### Human Coding

#### Sentiment

For the parent comments (ie, excluding replies, n=239), the “like” count ranged from 0 to 252. The mean “like” count was 5.6 (SD 19.9) and the median was 1 (IQR 0‐3). Of the 532 comments and replies, 73.8% (393/532) were relevant to NTPs. Approximately 64% of comments (250/393) expressed any NTP sentiment, with 42.8% of these (107/250) expressing pro-NTP sentiment, 38.8% (97/250) expressing anti-NTP sentiment, and 18.4% (46/250) expressing complex sentiment (Table 1).

**Table 1.** Frequencies of sentiment and content codes from human qualitative coding of YouTube comments including distribution of content code frequency by sentiment (n=393).

Code	Frequency, n (%)	Pro-NTP sentiment, n (%)	Anti-NTP sentiment, n (%)	Complex-NTP sentiment, n (%)
NTP^a^ sentiment	250 (63.6)	—^b^	—	—
Pro-NTP	107 (42.8^c^)	—	—	—
Anti-NTP	97 (38.8^c^)	—	—	—
Complex sentiment	46 (18.4^c^)	—	—	—
Content**^d^**				
Potential misinformation	108 (27.5)	66 (75.9)	12 (13.8)	9 (10.3)
e-cigarette^e^ use is safer than smoking	69 (17.6)	53 (80.3)	0 (0.0)	13 (19.7)
Personal experience	61 (15.5)	27 (56.3)	5 (10.4)	16 (33.3)
Health effects of e-cigarettes	50 (12.7)	7 (15.2)	26 (56.5)	13 (28.3)
Big tobacco	41 (10.4)	13 (65.0)	2 (10.0)	5 (25.0)
Addiction	39 (9.9)	6 (19.4)	16 (51.6)	9 (29.0)
Conspiracy	37 (9.4)	16 (69.6)	3 (13.0)	4 (17.4)
Marketing to youth	24 (8.7)	4 (15.4)	14 (53.9)	8 (30.8)
Distrust of medicine, research, or government	33 (8.4)	19 (90.5)	0 (0.0)	2 (9.5)
Flavors	27 (6.9)	12 (52.2)	6 (26.1)	5 (21.7)
E-cigarettes are as harmful as smoking	23 (5.9)	1 (4.8)	18 (85.7)	2 (9.5)
Blaming parents	22 (5.6)	1 (14.3)	3 (42.9)	3 (42.9)
Policy action or regulation	19 (4.8)	7 (53.9)	5 (38.5)	1 (7.7)
Personal smoking to e-cigarette use	19 (4.8)	12 (66.7)	0 (0.0)	6 (33.3)
EVALI^f^	13 (3.3)	5 (55.6)	1 (11.1)	3 (33.3)
Comparison to opioids or alcohol	11 (2.8)	2 (33.3)	1 (16.7)	3 (50.0)
More research is needed	9 (2.3)	3 (37.5)	1 (12.5)	4 (50.0)
Longing for JUUL	5 (1.3)	5 (100.0)	0 (0.0)	0 (0.0)
Learning from docuseries	3 (0.8)	0 (0.0)	1 (33.3)	2 (66.7)

a NTP: nicotine and tobacco product.

b Not applicable.

c Percent calculated out of total number expressing any sentiment (n=250).

d Codes are not mutually exclusive.

e e-cigarette: electronic cigarette

f EVALI: e-cigarette or vaping product use–associated lung injury.

#### Content

The most frequent content category was potential misinformation (27.5%, 108/393), followed by e-cigarette use is safer than smoking (17.6%, 69/393; eg, “Harm reduction matters more than people think. Cigarette smoking causes 8 million preventable deaths globally each year. A safer way to consume nicotine could save hundreds of millions of these deaths in the long run.”) and personal experience using e-cigarettes or JUUL (15.5%, 61/393; eg, “I was hooked on cigs for 35 years. I got an e-cig as a gift. Since then, I’ve been smoke-free for 13 years, nicotine-free for 10 years, and vape-free for 8 years. How many long-running smokers are never going to switch like I did because of what these jerks did to the vaping industry?”). Other frequently occurring content categories included health effects of e-cigarettes (12.7%, 50/393) and Big Tobacco (10.4%, 41/393; Table 1).

Examining the distribution of content code frequency by sentiment, approximately 76% (66/108) of comments coded as potential misinformation expressed pro-NTP sentiment. Similarly, the majority of comments coded as e-cigarette use is safer than smoking and personal experience expressed pro-NTP sentiment, although 33.3% (16/61) of comments coded as the latter expressed complex sentiment (Table 1). Almost all of these comments mentioned the poster using e-cigarettes but not using JUUL, such as “as someone who vaped for over a decade without any major health problems, I’m really glad I stayed away from Juul.”

Of note, while the majority of comments coded as e-cigarette use is safer than smoking were also coded as potential misinformation, approximately 27% (19/69) were not. These comments often used a personal anecdote (eg, “I quit smoking three years ago and my wife and I started using a Juul. Since then, we have taken up running, biking, kayaking—you name it—all thanks to quitting cigarettes.”), therefore were not considered potential misinformation. Similarly, about half of the comments coded as e-cigarettes are as harmful as smoking were not coded as potential misinformation (52.2%, 12/23). These comments acknowledged that more time is needed to determine the full health effects of e-cigarettes (eg, “As a registered nurse, I assisted in a lung cancer surgery. I can imagine that in 20 or more years, we start seeing the same health problems linked to vaping.”) or acknowledged the differences between the 2 products in terms of harms (eg, “Vapes take the lives of young people, while cigarettes take the lives of adults. Think about that.”).

#### Qualitative Analysis of Potential Misinformation Comments

Among the 108 comments coded as potential misinformation, qualitative analysis revealed 152 individual pieces of misinformation that were broadly grouped within 6 themes (Multimedia Appendix 2). The most frequent theme was that E-cigarette use is completely safe or much safer than smoking, found in 80 statements (Multimedia Appendix 2). Three distinct subthemes emerged; the first focused on declarative statements that e-cigarette use is much safer than smoking, which often included declarative language such as “definitive” (eg, “vapes are definitely 1000x a better alternative to classic Marlboros.”) or “far healthier” (eg, “It seems safer to say that they are much healthier than tobacco products.”). Statements often stated that e-cigarettes are 95% safer than smoking, an estimate that is not backed by concrete evidence [40]. Another subtheme was that e-cigarette use has saved millions of lives (n=20), considered potential misinformation due to the lack of evidence around the population-level harms versus benefits of e-cigarette use, as well as conflicting results from modeling studies [41,42]. A final subtheme was that E-cigarette use has no known harms or is never harmful, which often suggested that there are no harms associated with e-cigarette use with statements such as “no solid evidence suggesting vaping is significantly harmful in any way.”

The second misinformation theme, which appeared in 21 statements, was broadly centered on conspiracies, policies, or other types of misinformation. A recurring subtheme was conspiracy theories about the “take down” of e-cigarettes by various entities, including governmental agencies, professional health organizations, and Big Tobacco because the sale of e-cigarettes affected their profits or grant funding. Other subthemes included misinformation about the FDA’s actions related to JUUL and varied pieces of misinformation that occurred with less frequency and were not consistent with other themes or subthemes (Multimedia Appendix 2).

The third misinformation theme was that EVALI was caused only by THC (n=19). Statements in this category often incorrectly stated that all or 100% of EVALI cases were caused by THC or black-market products (eg, “All cases were linked to off-market products.”) or minimized the number of cases (eg, “Only a small number of individuals were affected by lung damage”). The fourth misinformation theme focused on health effects, benefits, and nicotine-related misinformation, which occurred in 19 statements and consisted of 3 subthemes. One subtheme consisted of exaggerations about the negative health effects of e-cigarettes or nicotine, which were often claims that have not yet been conclusively established by research. Other subthemes focused on the suggestion that nicotine is benign or is the same as caffeine and that nicotine or e-cigarettes have unproven or exaggerated health benefits, which were often statements that have not yet been conclusively established by research. The fifth misinformation theme was equating e-cigarettes with cigarettes, which was found in 10 statements and included 2 subcategories. The first, that e-cigarette use is the same as smoking, often implied that e-cigarettes and cigarettes are exactly the same, while the second, that the only harmful part of smoking cigarettes is the combustion, often left out the fact that nicotine is addictive and can be harmful to the developing adolescent brain. Finally, the sixth misinformation theme was e-cigarettes presented as a proven smoking cessation tool, which was found in 3 statements (Multimedia Appendix 2).

### Topic Modeling

#### Frequency Analysis

After removing common stop-words, the most frequent words in the cleaned dataset were “vaping” with 158 occurrences, “people” with 117 occurrences, “juul” with 114 occurrences, “kids” with 108 occurrences, and “smoking” with 91 occurrences. Other relevant frequently occurring terms were “tobacco,” “cigarettes,” “nicotine,” “bad,” and “health.” Refer to Multimedia Appendix 3 for the complete list of the 200 frequently occurring words.

#### Topics Covered in YouTube Comment Discussions

We initially identified 10 topics based on the most frequent terms associated with each topic. One topic (Topic 4) comprised predominantly non-English comments and was removed as irrelevant to the focus of our analysis, leaving 9 coherent topics. To assess robustness, we compared 3 modeling approaches: LDA, NMF, and BERTopic. NMF achieved the highest semantic coherence (0.48), followed by LDA (0.41) and BERTopic (0.36). However, NMF topics exhibited greater redundancy, and BERTopic produced several clusters dominated by function words. The 10-topic LDA model provided the best balance of interpretability, coherence, and exclusivity, and its themes aligned most closely with our qualitative coding framework. The 9 retained topics were qualitatively grouped into 4 thematic categories: comparisons with other drugs, mentions of government and pharmaceutical companies, the role of media and parents, and harms associated with nicotine and tobacco products (Table 2). The aggregated LDA weights of the most relevant terms across the 4 thematic groups are presented in Multimedia Appendix 4.

**Table 2.** Thematic categories of 9 topics and relevant terms of each topic as identified in the analysis of 532 YouTube comments.

Category	Relevant terms (Most Relevant Terms are in *italics*)
*Comparisons with other nicotine, tobacco, alcohol, and other products*	
Topic 5	Juul, *smoking, vaping, cigarettes*, people, documentary, really, long, bad, think, term, health
Topic 8	vaping, years, *nicotine*, believe, smoking, *juul, vape***,** much, *cigarettes***,** just, now, smoke, got, free
Topic 9	Kids,just, brand, vaping, *thc, vitamin,* people, market, *carts, vape, juices*, fact, used, juul
Topic 10	want, get, good, can, like, people, *alcohol*, problems, know, just, make, better.
*Mentions of Government and Pharma Companies*	
Topic 2	people, juul, *tobacco***,** can, *vape, big, health, companies***,** nicotine, *control*
Topic 7	vaping, tobacco, harmful, smoking, *people***,** product, health, smoke, bad, *government, lives,* used.
*Role of Media and Parents*	
Topic 1	*kids***,** now, just, people, juul, know, vape, vaping, smoking, don’t, stop, *parents, blame*
Topic 6	*kids***,** juul, don, quot, people, *media***,** just, vape, addicted, *social***,** got, even, *reason, cool*
*Harms associated with Nicotine and Tobacco Products*	
Topic 5	Juul, smoking, vaping, cigarettes, people, documentary, *really, long, bad, think, term, health*
Topic 3	vaping, years, juul, smoking, nicotine, tobacco, people, every, cigarettes, smoke, like, *life, lungs*
Topic 7	vaping, tobacco, *harmful,* smoking, people, product, *health,* smoke, *bad,* government, *lives, used*.

## Discussion

### Principal Findings

To the best of our knowledge, this is the first study to examine viewer reactions to the Netflix docuseries “Big Vape: The Rise and Fall of JUUL.” Human qualitative coding of comments and replies posted in response to the docuseries trailer and video clip on the Netflix YouTube channel found that most comments were relevant to NTPs, and that almost one-third of comments expressed potential misinformation about these products. Topic modeling found that the most frequent words highlighted central themes related to e-cigarette use, particularly focusing on JUUL and its association with youth. Finally, topic modeling found 9 relevant topics, roughly grouped into 4 thematic areas. Overall, our findings highlight the potential utility of analyzing YouTube comments in response to an e-cigarette–related docuseries to identify public perceptions of these products and potential misinformation about them.

To date, most analyses of e-cigarette–related content on social media have classified sentiment as positive (ie, pro-e-cigarette), neutral, and negative (ie, anti–e-cigarette) [43-45]. In the course of codebook development, we noted multiple comments where the author expressed pro–e-cigarette sentiment but anti-JUUL sentiment or vice versa. Thus, we included a complex sentiment code to capture these comments; almost 20% (46/250) of comments expressing sentiment were coded as complex. This finding underscores the importance of including more nuanced sentiment codes in future analyses of e-cigarette–related social media data, particularly when analyzing data related to specific products or policies. It also underscores the potential difficulty in using automated techniques to evaluate sentiment, as well as the need for public health professionals to develop survey items that can capture complex viewpoints and tailored health communication messages that acknowledge nuances in opinions about e-cigarette products.

Given that almost half of the comments that expressed NTP sentiment were pro-NTP, it is perhaps not surprising that the majority of comments in our most frequently coded content categories (potential misinformation, e-cigarette use is safer than smoking, and personal experience) were pro-NTP. However, it is notable that about one-third of personal experience comments expressed complex-NTP sentiment, a finding in contrast to previous studies of Twitter/X that found personal experience messages to be overwhelmingly positive [18,46]. This difference may be due in part to this previous work not classifying sentiment as complex, or it may be reflective of more nuanced positions held by e-cigarette users around support for JUUL or other specific products. Future research can build from this finding to further investigate the sentiment of e-cigarette users around NTPs, and public health professionals can use these findings to develop more nuanced health communication and health education efforts.

Previous studies examining a variety of health-related content on TikTok (ByteDance Ltd), Twitter, and YouTube have found health-related misinformation in approximately 5%‐15% of posts [47-50], and a study specifically examining e-cigarette–related content on Twitter/X found potential misinformation in 12.8% (377/2943) of posts [19]. Thus, we were surprised to find potential misinformation in over 25% (108/393) of comments in our sample. As some of the misinformation themes were specific to JUUL (eg, JUUL’s only mission was to stop adult cigarette smoking), it may be that the greater frequency of potential misinformation in our sample was due to the subject matter—the JUUL docuseries—of the videos the comments were posted. However, we also observed similarities in misinformation themes, particularly that e-cigarette use is safe or much safer than smoking, misinformation about EVALI, and exaggerations of the known harms of e-cigarette products, between our findings and previous work examining misinformation about e-cigarettes on Twitter/X [19,33]. We also found frequent mention of the claim that e-cigarettes are 95% safer than smoking cigarettes. As discussed by Eissenberg et al [40], this estimate is considered a “factoid,” or a statement repeated so often it is considered a fact without supporting evidence. These findings suggest the need for public health professionals to develop comprehensive health communication campaigns across social media platforms to address common misinformation themes, and for future research to examine ways in which e-cigarette misinformation may spread across social media platforms.

Analyses of viewer comments on other health-related docuseries and videos show both parallels and distinctions with our findings. For instance, comments on antivaccine videos on YouTube have similarly featured personal stories and emotionally charged discourse (ie, fear-based or toxic messaging) accompanied by claims widely recognized as misinformation [27]. However, sentiment in these discussions tended to be more distinct, whereas sentiment in our dataset was characterized by more complex views (ie, pro-e-cigarette but anti-JUUL or vice versa). In addition, while previous research on a family planning documentary has found that viewer discourse after viewing the content often centered on personal stories and expressions of learning [15], we found a greater emphasis on product-specific comparisons, brand-related narratives (eg, the “95% safer” factoid), corporate intent, and policy debates. These differences suggest that docuseries addressing commercially branded products with a recent history of regulatory action may elicit more polarized, misinformation-prone, and policy-focused engagement compared to those on other health topics.

A review of the literature on social media content analysis published in 2023 recommended that future research synthesize human qualitative coding and software-based methods [51], and our findings demonstrate the feasibility and benefits of this approach as related to social media perceptions of e-cigarettes. One benefit may be at the codebook development stage. For example, based on our initial human analysis of comments, we developed a code for blaming parents*,* but not one for comments that blamed the youth themselves. However, topic modeling revealed that the terms “blame” and “kids” appeared in Topic 1, suggesting that blaming youth or kids was a relevant theme. Similarly, while our human qualitative codebook had content codes for both EVALI and potential misinformation, we did not create a separate code for THC itself. However, topic modeling identified THC as a significant term in Topic 9, demonstrating the method’s ability to highlight themes that might have been overlooked in manual coding. Similarly, due to comments examined during the initial stages of codebook development, we anticipated that comparison to opioids or alcohol would be key content code, yet these only ended up being mentioned in less than 3% (11/393) of comments. Topic modeling found that alcohol was still discussed in Topic 10, but substances were not as prevalent in the larger dataset as initially expected.

In the given context, we can argue that with smaller datasets like in this study, the integration of topic modeling with human coding offers a useful and efficient method for uncovering both expected and unexpected themes. This approach combines the strengths of both data-driven insights and human contextual interpretation, enhancing the quality of the analysis. In future research, these methods can be applied to larger datasets, where the volume of data would make manual coding alone impractical. As dataset sizes grow, topic modeling can serve as a more robust foundation for identifying trends, while human coding remains essential for nuanced interpretation and validation. The success of this mixed approach in smaller datasets suggests its potential for scalability and greater efficiency in handling larger, more complex datasets.

### Limitations

We extracted all comments and replies 1 month after the docuseries release, so we did not assess comments or replies posted after this date. While this was due to our focus on initial reactions to the docuseries, this time frame means we potentially missed evolving discussions or delayed reactions. We also did not analyze comments and replies temporally (ie, from multiple time points) and thus cannot make conclusions about how comments or opinions may have evolved over time, nor did we examine associations between comment popularity (ie, “likes” and replies) and sentiment. It was also beyond the scope of this study to examine the accounts in our dataset; thus, we do not know if the comments were authored by humans or automated accounts (ie, “bots”) nor the demographic characteristics of the authors. Future research could examine both temporal trends of comments as well as demographics of accounts posting the comments. For the human qualitative coding, interpretation of the comments can be subjective, although we aimed to minimize subjectivity by double-coding all comments plus adjudication with a researcher experienced in coding social media data and using systematic procedures for codebook development and coding. For topic modeling, the small dataset size of this study may have been insufficient for topic identification and may have limited the generalizability of the results. It is expected that larger datasets would produce more robust patterns and results, and future research could assess if our topics hold with larger datasets. Another limitation is that the use of topic modeling was largely exploratory. We used it as a tool to generate themes without any prior assumptions, which makes it useful for discovery but also less suited for confirming hypotheses or generating precise categorizations. Thus, the results from this approach should be interpreted as preliminary and open to further refinement.

### Conclusions

Our analysis of YouTube comments and replies posted in response to the trailer and official video clip for the Netflix docuseries “Big Vape: The Rise and Fall of JUUL” highlights the potential utility of analyzing these comments to identify sentiment around these products and potential misinformation about them. It also highlights the benefits of integrating computational and human qualitative research to analyze social media perceptions of e-cigarettes. Given ongoing regulatory and legal proceedings related to JUUL and other e-cigarette products, as well as the continued emergence of new NTP products, it is vital for public health to develop educational materials about the current science regarding e-cigarettes. Public health professionals can use our findings to help develop tailored health communication messages to address common sentiment and misconceptions.

## Supplementary material

10.2196/76737 Multimedia Appendix 1 Codebook used for human qualitative coding of YouTube comments. Coders used the definitions and examples presented to code each comment for the presence or absence of each code, and codes were not mutually exclusive. All examples are modified to prevent reidentification.

10.2196/76737 Multimedia Appendix 2 Themes identified in qualitative analysis of YouTube 108 comments coded as potential misinformation that revealed 152 individual pieces of misinformation.

10.2196/76737 Multimedia Appendix 3 List of the top 200 most occurring terms in the dataset.

10.2196/76737 Multimedia Appendix 4 Aggregated latent Dirichlet allocation weights of the most relevant terms across the 4 thematic topic groups.

## Abbreviations

API: application programming interface
CDC: Centers for Disease Control and Prevention
e-cigarette: electronic cigarette
ENDS: electronic nicotine delivery system
EVALI: e-cigarette or vaping product use–associated lung injury
FDA: Food and Drug Administration
LDA: latent Dirichlet allocation
NMF: negative matrix factorization
NTP: nicotine and tobacco product
PMTA: premarket tobacco product application
RA: research assistant
SRQR: Standards for Reporting Qualitative Research
THC: tetrahydrocannabinol
