# Supplementary material for: Response to the Netflix Docuseries “Big Vape: The Rise and Fall of JUUL”: Mixed Methods Analysis of YouTube Comments Using Qualitative Coding and Topic Modeling
Source: JMIR Form Res. 2025 Sep 19;9:e76737. doi: 10.2196/76737 (PMC12448255; doi:10.2196/76737)
Supplement: Multimedia Appendix 1 [file formative-v9-e76737-s001.docx]

Appendix 1. Codebook used for human qualitative coding of YouTube comments. Coders used the definitions and examples presented to code each comment for the presence or absence of each code, and codes were not mutually exclusive. All examples are modified to prevent re-identification.

| **Code** | **Definition** | **Example** |
| --- | --- | --- |
| **Sentiment** |  |  |
| Pro NTP | Expresses pro-nicotine e-cigarette sentiment and/or pro-JUUL sentiment. | - Vaping has saved millions of lives. It will be viewed as one of the greatest health related inventions of all time. |
| Anti NTP | Expresses anti-e-cigarette sentiment and/or anti-JUUL sentiment. | - First the BBC and now Netflix? My roommate vapes and now I am seriously worried for her. |
| Complex e-cigarette | Expresses pro-e-cigarette but anti-JUUL sentiment or vice versa. Expresses complexity of evidence of harms or people choosing to use e-cigarettes or JUUL. | - Thanks to Juul, I quit smoking cigarettes. Yes, it took me a long time to stop using Juul, but I did it. Now I am free. Thank you Juul! Too bad that young people start using Juul. Their early promotion of Juul was wrong; I agree. |
| No sentiment | Not pro or anti; also not complexity. Instead, doesn’t really state any kind of sentiment about NTPs at all | - Why does the documentary make it sound like Juul invented the idea of vaping? |
| **Content** |  |  |
| Personal experience | Mentions personal experience with using e-cigarettes | - Been vaping six years now and no major issue I only vape 3ml but I don’t have anything majorly wrong with me. Except when I get a cold that's it. Sure I'll get something but I’m not worried. |
| Smoking to e-cigarettes | Mentions personal experience using e-cigarettes to quit smoking | - I smoked for 20 years, switched to Juul for 2 years and then quit Juul. I'm nicotine free now. So is Juul a scapegoat for all vaping or just being dissolved by big tobacco? I'm perplexed |
| Flavors | Mentions flavoring in JUUL or e-cigarettes | - IF you're switch from smoking it makes sense, the ideal solution is a standard system with three flavors (regular/menthol/vanilla) and that’s it. |
| Addiction | Mentions addiction to nicotine or to using e-cigarettes. Can be personal experience with addiction OR any mention of the product being addictive | - I have a love hate relationship with this product, this thing successfully took me off cigs but then just gave me a new, worse addiction one because you were now able to smoke anywhere, bathroom at work, at home etc - here was no downtime. It was to a point that once I even woke up I would hit the Juul…I felt like getting off of Juul was more difficult than if I was to stop smoking cigarettes. |
| Health effects | Mentions negative health effects of nicotine e-cigarettes or using JUUL | - Juuling was so much worse than cigarettes. Because there are virtually no restrictions where you can vape and Vaping was constant unlike cigarettes where you only smoke during breaks. Juul on the other hand made my heart weak and felt fatigued all the time. |
| More research | Mentions that more research is needed about e-cigarette use | - If there is any unseen damage by the unknown chemicals, it probably will surface in about 50 years of trials and research and I am not suggesting the use of either. |
| Longing | Mentions wishing they could still buy certain flavors of JUUL or products that are no longer on the market | - I wish I could still purchase Menthol Juul Pods in NYS. It's not a fruity flavor, it’s outrageous that lobbyists for Big Tobacco have so much power. STRAIGHT-UP attack on Juul. It’s heinous. |
| Marketing to youth | Mentions marketing of JUUL/e-cigarette products to youth | - My takeaway from this documentary is: marketing to stupid teenagers ruined a good opportunity to get people off of tobacco. |
| E-cigarettes are safer | Says that e-cigarettes are safer and more useful than alternative methods of ingesting nicotine and thus less harmful than cigarettes | - Idk how you can label it a “fail” When millions of people still do it. And ask your doctor they will still tell you anything you put in your lungs is dangerous but it’s still better than cigs because no there is no tar. |
| E-cigarettes are as harmful as smoking | E-cigarette use is as harmful or worse than smoking cigarettes | - Juuling was way worse than cigarettes. Because there are essentially no restrictions where you can vape and the constant vaping unlike cigarettes where you only smoke during smoke breaks. Juul on the other hand made my heart weak and felt tired all the time. Glad that I’ve quit it |
| Potential misinformation | States an unverifiable claim or a scientifically invalid claim about e-cigarettes or other NTPs. States a claim about policy that is not accurate. | - Nicotine isn't harmful ya idiot. |
| Blaming parents | Mentions that parents should shoulder some of the blame for their kids using e-cigarettes | - Just finished the series- parents are also to blame- how do middle schoolers get money to buy Juuls? Who is watching these kids???? Shame on the Silicon Valley tech machine that pursues money at all costs and shame on parents for not knowing what their kids are doing, watching etc and shame on children for being sheep- nothing should go in your lungs but air. |
| Learning | Mentions learning something from the docuseries | - Very nice documentary. As an European vaper I had no idea this has been going on in the United States for the past 7 years, it's so mind blowing. |
| Policy action or regulation | Explicit policy action or regulation of e-cigarettes such as flavor restrictions and retail access. Not hypothetical. | - I remember when they operated under the cover of Covid outlawing it in Massachusetts. Then when the time was right, they reintroduced flavored vapes with a 300% increase in taxes. The problem is, we all just drove 35 minutes to Connecticut to give Connecticut our tax dollars instead. What a joke our government is. |
| Big Tobacco | Mentions Big Tobacco or tobacco lobbying | - Big tobacco succeeds again |
| Conspiracy | Mentions a conspiracy with the government or a conspiracy about diseases from e-cigarettes. | - I am not a paranoid conspiracy theory type person, but documentaries like this one, I can 100% guarantee are somehow sponsored by big tobacco. |
| Comparison to opioids or alcohol | Mentions a comparison of JUUL/e-cigarettes to opioids or alcohol | - So the FDA were content letting Oxy go unchecked for years but got upset about e cigs. |
| Distrust of medicine, research, or government | Indicates a distrust of science, medicine, research, and/or government | - I remember when they operated under the cover of Covid outlawing it in Massachusetts. Then when the time was right, they reintroduced‚ flavored vapes with a 300% tax increase. The problem is, we all just drove 35 minutes to Connecticut to give Connecticut our tax dollars instead. What a joke our government is. |
| EVALI | Mentions EVALI or vaping lung disease that sounds like EVALI (e.g. respiratory illness) | - The 'collapsed lungs' or popcorn lungs, or any lung damage caused in the handful of users who experienced it, all of the cases were linked to non- market products made with Vitamin-E acetate, which they of course don't use in brand name e-juices. In fact, that ingredient was almost exclusively used in THC carts from the black market. |
