# Supplementary material for: Response to the Netflix Docuseries “Big Vape: The Rise and Fall of JUUL”: Mixed Methods Analysis of YouTube Comments Using Qualitative Coding and Topic Modeling
Source: JMIR Form Res. 2025 Sep 19;9:e76737. doi: 10.2196/76737 (PMC12448255; doi:10.2196/76737)
Supplement: Multimedia Appendix 2 [file formative-v9-e76737-s002.docx]

Appendix 2. Themes identified in qualitative analysis of YouTube 108 comments coded as potential misinformation that revealed 152 individual pieces of misinformation

| **Misinformation Themes and Subthemes** | **Total Number of Statements** | **Rationale for Inclusion as Misinformation** | **Examples** |
| --- | --- | --- | --- |
| Theme 1: E-cigarette use is completely safe or much safer than smoking (n=80) | | | |
| Subtheme 1:  E-cigarette use is much safer than smoking | 44 | There is evidence that, for established long-term smokers, switching to e-cigarettes may be healthier, but research continues to identify harms related to e-cigarette use, such as higher risk of heart failure [1]. It is problematic for youth to think that e-cigarettes are not harmful [2], and the “e-cigarettes are 95% safer” statement has been found to lack evidence [3]. | - Vaping is 95% safer than smoking! If you think otherwise, you're mistaken! These are the facts - Every study conducted so far shows that vaping is a healthier alternative to smoking. |
| Subtheme 2:  E-cigarette use has saved millions of lives | 20 | While some modeling studies suggest e-cigarette use could save millions of lives, other modeling studies suggest that e-cigarette use will lead to more lives lost [4,5]. | - Vaping has been a major positive innovation, saving millions of lives. - It’s a significant innovation that has saved countless lives. |
| Subtheme 3:  E-cigarette use has no known harms or is not harmful | 16 | Some known harms of e-cigarettes include popcorn lung, EVALI, and explosions [6–8]. There are also specific harms associated with young people, such as nicotine affecting brain development [9]. | - To this day, vaping has not been proven to be harmful. - There’s no research that supports the idea that vaping is harmful in any significant way. |
| Theme 2: Conspiracies, policy-related, and other misinformation (n=21) | | | |
| Subtheme 1: Conspiracy theories about the “take down” of  e-cigarettes by various entities | 10 | There is no evidence that governments or researchers want to abolish e-cigarettes for financial reasons, or that tobacco companies have tried to “take down” e-cigarette manufacturers. | - The government opposes vaping because it cuts into tobacco tax revenue. - The American Heart Association gets all of its funding from anti-tobacco efforts. |
| Subtheme 2: Other e-cigarette, JUUL, or policy-related misinformation | 9 | Specific pieces of misinformation were varied. | - Her heart felt weak because it contained 50 percent nicotine, which is five times more than a cigarette. - All the exaggerated claims about "popcorn lung" are specifically tied to Juul. - I remember when they secretly banned vaping in Massachusetts under the guise of Covid restrictions. |
| Subtheme 3: The FDA quickly banned flavored JUUL pods | 2 | Although the FDA did ban mint and fruit flavored JUUL pods, this did not happen until June 2022, about two years after the EVALI outbreak [10]. In November 2019, anticipating a ban, JUUL ended sales of mint pods voluntarily [11]. | - They banned mint-flavored Juul pods without any proof of harm. - Mint-flavored Juul pods were banned in just a month. |
| Theme 3: EVALI-related misinformation (n=19) | | | |
| EVALI was caused only by THC | 19 | Although the majority of EVALI cases were linked to black market THC products, a small subset were linked to e-cigarettes [12]. | - It wasn’t Juul that harmed young people's lungs; it was the additives in the vape liquids that caused the issue. A few people had coincidental lung problems. - Only about 18 people in the entire country got sick from vaping illegal cannabis cartridges and Covid. |
| Theme 4: Health effects, benefits, and nicotine-related misinformation (n=19) | | | |
| Subtheme 1: Exaggeration about the negative health effects of e-cigarettes or nicotine. | 9 | While e-cigarettes are not without harm, there is evidence that, for established long-term smokers, switching to e-cigarettes may reduce exposure to toxicants [13]. Other comments in this category included exaggerations of the negative aspects of e-cigarettes, mostly claims that have not yet been established by research. | - Just wait until people hear that vaping causes shrinkage of their balls. - Using Juul was far worse than smoking cigarettes. |
| Subtheme 2: Nicotine is benign and/or is the same as caffeine. | 7 | This is a myth that is perpetuated by the tobacco industry; JUUL co-founder said it in 2018 [14].) Nicotine has negative effects on the cardiovascular system and may affect brain chemistry [15]. | - Nicotine is a relatively harmless substance, no worse than caffeine. - Nicotine itself is not a harmful ingredient. |
| Subtheme 3: Nicotine/ e-cigarettes have unproven (or exaggerated) health benefits. | 3 | There is no concrete evidence that nicotine is beneficial. While some researchers have suggested it could be [16], research is still needed to evaluate this claim. | - Nicotine is a mild cognitive enhancer, beneficial especially for older individuals. - Some studies suggest nicotine has positive effects on brain function. |
| Theme 5: Equating e-cigarettes with cigarettes (n=10) | | | |
| Subtheme 1:  E-cigarette use is the same as smoking | 7 | While e-cigarette use is associated with several harms, some of these are different than those associated with smoking cigarettes [17]. | - Vaping is just smoking repackaged in a new form. - Vaping and smoking are essentially the same thing, just different forms. |
| Subtheme 2: The only harmful part of smoking cigarettes is the combustion | 3 | It is not just the combustion in cigarettes that is harmful. While cigarette combustion does include almost 70 chemicals that are known carcinogens, other aspects of cigarettes (like the nicotine) are also harmful [18]. Also, while it is true that e-cigarettes do not involve combustion, the idea that the only harmful part of cigarettes is combustion leads to the “e-cigarettes are safe” narrative. | - The danger lies in the combustion (smoke), not the vaping process. - Combustion is what makes cigarettes harmful. |
| Theme 6: E-cigarettes presented as a proven smoking cessation tool (n=3) | | | |
| JUUL’s mission was primarily to end smoking or e-cigarettes are a smoking cessation tool. | 3 | While “ending smoking” by switching adult smokers to e-cigarettes was touted as their primary mission, evidence suggests that JUUL marketed to youth [19].  E-cigarettes are not one of the quit smoking medicines approved by the FDA [20]. | - Juul is effective in helping people quit smoking and eventually stop altogether. - They weren’t marketing to kids; they were trying to prevent children from buying their products. |

References:

1. American College of Cardiology. Study Links E-Cigarette Use with Higher Risk of Heart Failure - American College of Cardiology. 2024. Available from: https://www.acc.org/About-ACC/Press-Releases/2024/04/01/21/51/study-links-e-cigarette-use-with-higher-risk-of-heart-failure [accessed Jul 9, 2024]

2. Li W, Kalan ME, Kondracki AJ, Gautam P, Jebai R, Osibogun O. Longitudinal impact of perceived harm and addiction on e-cigarette initiation among tobacco-naïve youth: Population Assessment of Tobacco and Health study (Waves 1–5). Public Health 2024 May 1;230:52–58. doi: 10.1016/j.puhe.2024.02.021

3. Eissenberg T, Bhatnagar A, Chapman S, Jordt S-E, Shihadeh A, Soule EK. Invalidity of an oft-cited estimate of the relative harms of electronic cigarettes. Am J Public Health American Public Health Association; 2020 Feb;110(2):161–162. PMID:31913680

4. Chu K, Shensa A, Colditz J, Sidani J, Hoffman B, Sinclair D, Krauland M, Primack B. Integrating social dynamics into modeling cigarette and e-cigarette use. Health Educ Behav 2020;47(2):191–201. doi: 10.1177/1090198119876242

5. Kalkhoran S, Glantz SA. Modeling the health effects of expanding e-cigarette sales in the United States and United Kingdom. JAMA Intern Med 2015 Oct;175(10):1671. PMID:26322924

6. American Lung Association. Popcorn Lung: A Dangerous Risk of Flavored E-Cigarettes. 2016. Available from: https://www.lung.org/blog/popcorn-lung-risk-ecigs [accessed Jul 7, 2024]

7. Edwards E. Vape pen explodes, shattering teen’s jaw amid rising concerns over batteries. NBC News. 2019. Available from: https://www.nbcnews.com/health/health-news/vape-pen-explodes-shattering-teen-s-jaw-amid-rising-concerns-n1018741 [accessed Jul 7, 2024]

8. E-cigarette, or Vaping Product, Use Associated Lung Injury (EVALI). Yale Med. 2024. Available from: https://www.yalemedicine.org/conditions/evali [accessed Jul 7, 2024]

9. CDC. E-Cigarette Use Among Youth. Smok Tob Use. 2024. Available from: https://www.cdc.gov/tobacco/e-cigarettes/youth.html [accessed Jul 5, 2024]

10. MacMillan C. The Juul E-Cigarette Ban: Will It Make a Difference? Yale Med. 2022. Available from: https://www.yalemedicine.org/news/juul-e-cigarette-ban [accessed Jul 9, 2024]

11. Kaplan S. Juul Ends E-Cigarette Sales of Mint-Flavored Pods. N Y Times 2019 Nov 7; Available from: https://www.nytimes.com/2019/11/07/health/vaping-juul-mint-flavors.html [accessed Jul 9, 2024]

12. Rebuli ME, Rose JJ, Noël A, Croft DP, Benowitz NL, Cohen AH, Goniewicz ML, Larsen BT, Leigh N, McGraw MD, Melzer AC, Penn AL, Rahman I, Upson D, Crotty Alexander LE, Ewart G, Jaspers I, Jordt SE, Kligerman S, Loughlin CE, McConnell R, Neptune ER, Nguyen TB, Pinkerton KE, Witek TJ. The E-cigarette or Vaping Product Use–Associated Lung Injury Epidemic: Pathogenesis, Management, and Future Directions: An Official American Thoracic Society Workshop Report. Ann Am Thorac Soc 2023;20(1):1–17. PMID:36584985

13. National Academies of Sciences, Engineering, and Medicine, Health and Medicine Division, Board on Population Health and Public Health Practice, Committee on the Review of the Health Effects of Electronic Nicotine Delivery Systems. Public Health Consequences of E-Cigarettes. Eaton DL, Kwan LY, Stratton K, editors. Washington (DC): National Academies Press (US); 2018. PMID:29894118ISBN:978-0-309-46834-3

14. Becker R. Why Big Tobacco and Big Vape love comparing nicotine to caffeine. The Verge 2019 Apr 26; Available from: https://www.theverge.com/2019/4/26/18513312/vape-tobacco-big-companies-nicotine-caffeine-comparison-drugs-chemicals [accessed Jul 9, 2024]

15. King A. Is nicotine bad for long-term health? Scientists aren’t sure yet. Nature 2023 Jun 7;618(7964):S10–S11. doi: 10.1038/d41586-023-01840-1

16. Nop O, Senft Miller A, Culver H, Makarewicz J, Dumas JA. Nicotine and Cognition in Cognitively Normal Older Adults. Front Aging Neurosci 2021 May 5;13:640674. PMID:34025390

17. Thomme GV. Does vaping cause lung cancer? MD Anderson Cancer Cent. 2024. Available from: https://www.mdanderson.org/cancerwise/does-vaping-cause-lung-cancer.h00-159694389.html [accessed Jul 9, 2024]

18. American Lung Association. What’s In a Cigarette? 2024. Available from: https://www.lung.org/quit-smoking/smoking-facts/whats-in-a-cigarette [accessed Jul 9, 2024]

19. NY State Attorney General. Attorney General James Secures $462 Million from JUUL for Its Role in the Youth Vaping Epidemic. 2023. Available from: https://ag.ny.gov/press-release/2023/attorney-general-james-secures-462-million-juul-its-role-youth-vaping-epidemic [accessed Nov 20, 2024]

20. Centers for Disease Control and Prevention. Quit Smoking Medicines. Tips Former Smokers. 2023. Available from: https://www.cdc.gov/tobacco/campaign/tips/quit-smoking/quit-smoking-medications/how-to-use-quit-smoking-medicines/index.html [accessed Nov 20, 2024]
