# Supplementary material for: Response to the Netflix Docuseries “Big Vape: The Rise and Fall of JUUL”: Mixed Methods Analysis of YouTube Comments Using Qualitative Coding and Topic Modeling
Source: JMIR Form Res. 2025 Sep 19;9:e76737. doi: 10.2196/76737 (PMC12448255; doi:10.2196/76737)
Supplement: Multimedia Appendix 3 [file formative-v9-e76737-s003.docx]

Appendix A: List of the Top 200 Most Occurring Terms in The Dataset

| Terms | Frequency |
| --- | --- |
| vaping | 158 |
| people | 117 |
| juul | 114 |
| kids | 108 |
| smoking | 91 |
| vape | 85 |
| like | 80 |
| tobacco | 71 |
| cigarettes | 67 |
| nicotine | 60 |
| get | 54 |
| still | 53 |
| big | 49 |
| years | 49 |
| even | 47 |
| documentary | 44 |
| vapes | 44 |
| bad | 44 |
| health | 44 |
| smoke | 44 |
| also | 43 |
| never | 37 |
| know | 36 |
| one | 36 |
| much | 36 |
| want | 35 |
| good | 35 |
| long | 32 |
| stop | 30 |
| made | 30 |
| harmful | 30 |
| got | 30 |
| really | 30 |
| lol | 29 |
| use | 29 |
| better | 29 |
| used | 28 |
| companies | 27 |
| lives | 27 |
| products | 26 |
| way | 26 |
| say | 26 |
| make | 25 |
| time | 25 |
| many | 25 |
| think | 25 |
| lung | 25 |
| something | 24 |
| millions | 23 |
| lungs | 23 |
| well | 23 |
| effects | 23 |
| less | 23 |
| industry | 23 |
| government | 22 |
| thc | 22 |
| addicted | 22 |
| problems | 22 |
| healthy | 22 |
| juuls | 22 |
| going | 21 |
| market | 21 |
| every | 21 |
| thing | 21 |
| cool | 20 |
| saved | 20 |
| believe | 19 |
| research | 19 |
| product | 19 |
| actually | 19 |
| doesn | 19 |
| quit | 19 |
| vitamin | 18 |
| wrong | 18 |
| around | 18 |
| parents | 18 |
| problem | 17 |
| hate | 17 |
| money | 17 |
| fact | 17 |
| media | 17 |
| alcohol | 16 |
| smokers | 16 |
| need | 16 |
| reason | 16 |
| makes | 16 |
| since | 16 |
| far | 16 |
| worse | 16 |
| term | 16 |
| high | 16 |
| anything | 16 |
| using | 15 |
| netflix | 15 |
| idea | 15 |
| right | 15 |
| nic | 15 |
| social | 15 |
| whole | 15 |
| day | 15 |
| nothing | 15 |
| significant | 15 |
| yet | 15 |
| things | 15 |
| cigarette | 15 |
| trying | 15 |
| started | 15 |
| getting | 15 |
| brand | 15 |
| major | 14 |
| reply | 14 |
| non | 14 |
| either | 14 |
| back | 14 |
| drugs | 14 |
| blame | 14 |
| teens | 14 |
| known | 13 |
| company | 13 |
| show | 13 |
| point | 13 |
| done | 13 |
| marketing | 13 |
| little | 13 |
| went | 13 |
| though | 13 |
| away | 13 |
| take | 13 |
| without | 13 |
| saying | 13 |
| let | 13 |
| acetate | 13 |
| put | 13 |
| always | 13 |
| cancer | 13 |
| isn | 13 |
| hard | 13 |
| vapor | 12 |
| instead | 12 |
| another | 12 |
| smoked | 12 |
| juice | 12 |
| sure | 12 |
| love | 12 |
| young | 12 |
| ingredient | 12 |
| everyone | 12 |
| lot | 12 |
| dont | 12 |
| hell | 12 |
| carts | 12 |
| stupid | 11 |
| new | 11 |
| school | 11 |
| dangerous | 11 |
| watching | 11 |
| feel | 11 |
| remember | 11 |
| free | 11 |
| life | 11 |
| end | 11 |
| care | 11 |
| great | 11 |
| date | 11 |
| buy | 11 |
| tell | 11 |
| studies | 11 |
| said | 11 |
| didn | 11 |
| seen | 11 |
| creates | 11 |
| evidence | 11 |
| shows | 11 |
| damage | 11 |
| control | 10 |
| year | 10 |
| teenagers | 10 |
| public | 10 |
| issue | 10 |
| comment | 10 |
| safer | 10 |
| different | 10 |
| dumb | 10 |
| com | 10 |
| name | 10 |
| study | 10 |
| addictive | 10 |
| course | 10 |
| weed | 10 |
| cause | 10 |
| anti | 10 |
| find | 10 |
| yes | 10 |
| maybe | 10 |
| drink | 10 |
| someone | 10 |
| agree | 10 |
| children | 10 |
| already | 10 |
| attention | 9 |
