# Supplementary figures and images for: Response to the Netflix Docuseries “Big Vape: The Rise and Fall of JUUL”: Mixed Methods Analysis of YouTube Comments Using Qualitative Coding and Topic Modeling
Source: JMIR Form Res. 2025 Sep 19;9:e76737. doi: 10.2196/76737 (PMC12448255; doi:10.2196/76737)

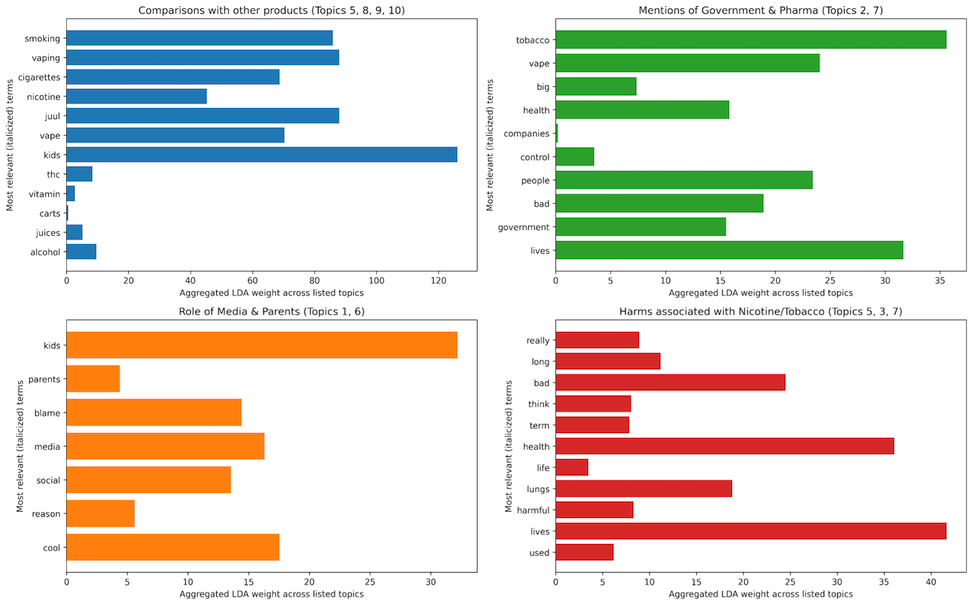

Supplement: Multimedia Appendix 4 [file formative-v9-e76737-s004.png]
